# Cheap Learning

**Published:** 1897-06

**Authors:** 


CHEAP LEARNING.—Dr. Orr, of Terrell, Tex., in this issue has an excellent article on the neglect of higher medical education, and incidentally scores the State for giving a medical education free. We believe in education;—a good, plain rudimentary English education, at least, should be given free to every child. Indeed, it ought to be compulsory; but when that is done the State has done its duty, and many persons hold with us, and with Dr. Orr, that it is superrogation on the part of the State to set a man up in business free of charge; that a medical education is a means of livelihood; it is a capital on which to operate to make a living, and the State should not tax its citizens to thus set up in business any man's son. This, however, is not the whole extent of the evil. Many persons think that a profession is a *sinecure*, a means of beating work, of obviating manual labor, something that most boys—farm boys especially, are not fond of; and hence, when a medical education is offered free, it is a temptation not to be resisted by the plow-boy and the smith's apprentice. In other words, temptation—inducements—are offered to those to study medicine who ought never to attempt it; boys who can never make doctors with all the schooling in the world; and hence the standard of medical education is necessarily kept low. Dr. Orr, as will be seen, points out that to compete with this free system—and there are those who think competition necessary, that there are not schools enough—it is necessary to make very low fees by those schools that are run by the professors for what's in them.

This leads to the next step: no education at all, but a diploma granted without even the form of attendance by some so-called schools, and finally not even a college building,—a charter only, granted by the State, and a diploma—the "Texas Health College," at Mound, Texas, for instance.

Incidentally, the doctor points out as a potent factor in keeping medical education to the lowest notch the existence of boards of medical examiners appointed by the district judges, who, knowing nothing of the applicant's qualifications to be an examiner, and caring less, make the appointments for political or social or personal reasons; hence, some of them are boards for revenue only. It is not a jest that there are boards in Texas thus appointed, who grant license while the train waits. We were informed at the late meeting at Paris, by a physician of repute, that he was witness to a case of the kind—that being in a saloon when the train arrived, two countrymen came in with two of the medical examiners for that district, and while the drinks were being mixed each examiner asked each man a single question, and then filled in the name of the applicants on forms which they carried in their pocket, filled out—all but the name of the victim—and signed. This occurrence alone ought to be sufficient to show how important it is that an examining board should only be appointed by the governor from a list of names of suitable men submitted by the organized medical profession. The custom as it now prevails, cited above, defeats the ends of any legislation to suppress quackery, for it legalizes (?) the very quacks sought to be excluded by *examination*. We are informed that one board has solicitors out amongst first-course students, offering a discount on "lots of five," the fee being $15 —the board will "knock off" if five or more present themselves. Was there ever such a condition of affairs tolerated in any other State? And still the profession pleads in vain for a medical act which will remedy this crying evil.

